# Relationship between the immune microenvironment of different locations in a primary tumour and clinical outcomes of oesophageal squamous cell carcinoma

**DOI:** 10.1038/s41416-019-0622-3

**Published:** 2019-11-25

**Authors:** Ken Hatogai, Satoshi Fujii, Shigehisa Kitano, Takashi Kojima, Hiroyuki Daiko, Takayuki Yoshino, Atsushi Ohtsu, Yuichi Takiguchi, Toshihiko Doi, Atsushi Ochiai

**Affiliations:** 1 Division of Pathology, Exploratory Oncology Research & Clinical Trial Center, National Cancer Center, Kashiwa, Chiba Japan; 2 Department of Gastroenterology and Gastrointestinal Oncology, National Cancer Center Hospital East, Kashiwa, Chiba Japan; 3 Department of Medical Oncology, Graduate School of Medicine, Chiba University, Chuo-ku, Chiba Japan; 4 Department of Experimental Therapeutics, Exploratory Oncology Research & Clinical Trial Center, National Cancer Center, Tokyo, Japan; 5 Department of Esophageal Surgery, National Cancer Center Hospital East, Kashiwa, Chiba Japan

**Keywords:** Oesophageal cancer, Tumour immunology

## Abstract

**Background:**

Tumour microenvironments can differ according to intratumoural locations. We investigated the immune status at different locations in primary tumours and its clinical significance in oesophageal squamous cell carcinoma (ESCC).

**Methods:**

The number of CD8^+^ tumour-infiltrating immune cells (TIICs) and PD-1^+^ TIICs, and PD-L1 expression on tumour cells (PD-L1_TC_) were immunohistochemically examined in the surface (Surf), centre (Cent) and invasive front (Inv) of tumours surgically resected from 192 patients with ESCC.

**Results:**

The PD-L1^+^ rate was lower in Inv than in Cent (12.0% vs. 18.2%, *P* = 0.012), although the numbers of CD8^+^ TIICs and PD-1^+^ TIICs were comparable among intratumoural locations. High numbers of CD8^+^ and PD-1^+^ TIICs and positive PD-L1_TC_ were related to better overall survival (OS) only in Surf and Cent (CD8: *P* = 0.012 in Surf, 0.018 in Cent, and 0.165 in Inv; PD-1: *P* = 0.028 in Surf, 0.021 in Cent, and 0.208 in Inv; and PD-L1: 0.044 in Surf, 0.026 in Cent, and 0.718 in Inv). Positive PD-L1_TC_ in Surf and/or Cent but not in Inv demonstrated a strong tendency toward better OS (*P* = 0.053).

**Conclusions:**

Immune microenvironments according to the intratumoural location have different effects on the survival of patients with ESCC.

## Introduction

The emergence of immune checkpoint inhibitors has been revolutionising cancer therapeutics, from therapy for melanoma and lung cancer to that of various other cancer types.^1^ Regarding gastrointestinal cancers, PD-1 inhibitors have been approved as one of the standard therapies for gastric cancer and microsatellite instability-high or mismatch repair-deficient colorectal cancer and are currently under development as therapy in the field of oesophageal squamous cell carcinoma (ESCC), the standard therapy for which has remained unchanged for more than a decade.^2^ PD-L1 expression is correlated with higher efficacy of therapy with immune checkpoint inhibitors in several cancer types, and pembrolizumab shows promising efficacy in patients with PD-L1^+^ ESCC.^3,4^ Furthermore, PD-L1 expression is related to the number of tumour-infiltrating immune cells (TIICs) as well as survival outcomes, according to recent reports, including our previous one on ESCC; this suggests that PD-L1 expression reflects an inflamed state of the tumour microenvironment.^5–7^ Therefore, it is important to determine the status of PD-L1 expression as a prognostic biomarker as well as a predictive biomarker for immune checkpoint inhibitors in clinical trials and future clinical practice.

The surface of the oesophageal lumen is exposed to various stimuli that may cause inflammation, such as oral intake of substances including alcohol, oral microbiota, and gastroesophageal reflux. In addition, gastrointestinal cancers originate from the mucosal epithelial layer and invade into deeper layers, such as the submucosa, muscle and further, as they progress.^8^ Analysis of gene alteration in separate areas of the same primary tumour has revealed that intratumoural genetic and epigenetic heterogeneity is common in ESCC, indicating clonal evolution in tumours.^9,10^ Therefore, the immune status of the tumour microenvironment may differ according to the location, even in a primary tumour. However, to the best of our knowledge, no studies have investigated intratumoural heterogeneity in terms of the immune status of the tumour microenvironment, including PD-L1 expression as well as the infiltration of immune cells, in ESCC. Besides, the relationship between such a difference in the immune status of the tumour microenvironment based on location and its clinical impact is unknown.

Pathological assessment in patients with ESCC is often performed using tumour samples obtained from the surface of primary tumours in the oesophagus with forceps, as a biopsy through an endoscope, which is also applied to assess the PD-L1 expression status immunohistochemically. The heterogeneity of PD-L1 expression in primary tumours has been studied by several methods in lung cancer, for which inconsistent results have been obtained.^11,12^ Elucidating intratumoural heterogeneity of the immune status of the tumour microenvironment could be helpful when considering the appropriateness of using endoscopically obtained biopsy samples for PD-L1 assessment in ESCC.

We conducted this retrospective immune-related biomarker study to elucidate intratumoural heterogeneity in terms of infiltration of immune cells and PD-L1 expression in different locations in primary tumours of ESCC and investigated the relationships between the immune microenvironment according to the intratumoural location and survival outcome.

## Materials and methods

### Patients

Among the 318 patients who underwent curative surgical resection of ESCC with no prior therapy between 2000 and 2011 at the National Cancer Center Hospital East, Kashiwa, Japan, 192 were consecutively enrolled in this study based on the following selection criteria: (i) pathological T factor of at least T2 according to the TNM classification^8^ for evaluating the difference of the three locations described below and (ii) a sufficient amount of formalin-fixed paraffin-embedded surgically resected tissue sample available for PD-L1 immunohistochemistry (IHC) evaluation for all of the three locations described below. Clinical and pathological information, including the pathological report for each subject, was collected from medical records. The study protocol was approved by the institutional review board of the National Cancer Center and was conducted in accordance with ethical guidelines, including the Declaration of Helsinki. The study was also conducted in accordance with the guidelines of the REporting recommendations for tumour MARKer prognostic studies (REMARK).^13^ Written informed consent was obtained from all participants included in this study.

### Immunohistochemistry

We first examined haematoxylin and eosin (H&E) stained slides of the archived primary tumours. Then, using a manual tissue arrayer (Azumaya Ika Kikai, Tokyo, Japan), we obtained 2.0-mm-diameter tumour cores from the surface (Surf), which is just below the surface of the tumour without necrotic tissue; the centre (Cent), which is an area within 3 mm of the vertical centre of the tumour; and the invasive front (Inv), which is within 3 mm from the invasive margin of the tumour side. These cores were assembled in a tissue microarray (TMA) format, and paraffin-embedded TMA blocks were then cut into 4-µm sections and placed on silicon-coated slides for IHC staining.

To assess PD-L1 expression on tumour cells (PD-L1_TC_), clone E1L3N, which was reported to show staining concordant to that of FDA-approved companion diagnostics for PD-L1 expression, was used.^14^ CD8 and PD-1 expression on TIICs were assessed based on our previous report, which demonstrated the significance of those types of TIICs for survival.^7^ In Supplementary Table 1, the primary antibodies used for IHC are described. For CD8 antibodies, which were optimised for autostainers, IHC was performed using ready-to-use antibodies and the fully automated Ventana Benchmark ULTRA platform (Ventana, Tucson, AZ, USA), in accordance with the manufacturer’s instructions. For PD-1 and PD-L1, IHC was performed manually. Briefly, for PD-1 and PD-L1, the slides were dewaxed and rehydrated in distilled water, and endogenous peroxidase activity was then blocked by immersion in 3% hydrogen peroxide in methanol for 10 min. After antigen retrieval, the slides were incubated overnight at 4 °C with the primary antibody. The slides were then further incubated with anti-mouse secondary antibodies (EnVision + System-HRP Labeled Polymer Anti-mouse, Dako, Tokyo, Japan) for PD-1 or anti-rabbit secondary antibodies (EnVision + System-HRP Labeled Polymer Anti-rabbit, Dako) for PD-L1, and staining was detected using a standard diaminobenzidine procedure with a fixed revelation time of five minutes. Finally, the sections were counterstained with haematoxylin.

### Evaluation of protein expression using IHC

After IHC, the slides were scanned with ×40 resolution, and the microscopic images were imported as digital photo files (NDPI format) using the NanoZoomer Digital Pathology (NDP) system (NanoZoomer2.0-HT C9600-02, Hamamatsu Photonics, Hamamatsu, Japan). The pathological evaluations were performed by two observers (including a gastrointestinal expert pathologist) who were blinded to clinical data. PD-L1_TC_ was defined as the presence of ≥1% tumour cells detected with membrane staining, as reported previously.^7^

To quantitatively evaluate each TIIC type, the entire tumour core was reviewed using the NDP view at a magnification of ×20, and four independent areas with a size of 0.0625 mm^2^, containing the highest number of TIICs in the tumour nest, were selected.^15,16^ The tumour areas were determined based on the IHC slides and the corresponding H&E staining of the adjacent serial section. After identifying the number of TIICs in each selected area by manual eye counting using the NDP view at a magnification of ×40, the numbers of respective TIICs per square millimetre were calculated from the total number in the four selected areas.

### Statistical analysis

For CD8 and PD-1, the numbers of TIICs in each intratumoural location were compared using the Mann–Whitney U test, and the correlation according to each location was evaluated using Spearman’s correlation coefficient ρ. For PD-L1, the positive rate in each intratumoural location was compared using McNemar’s test. Using the Kaplan–Meier method, we estimated overall survival (OS), and using the log-rank test, we categorised them according to positivity or negativity for PD-L1_TC_. Then, we compared OS levels of two groups divided by the median for TIICs. Hazard ratios (HRs) adjusted for clinicopathological characteristics, described in Table 1, were also reported using the multivariate Cox proportional hazard model (adjusted HR). IBM SPSS Statistics 20 (IBM Japan Ltd., Tokyo, Japan) was used to perform all statistical analyses. All *P* values are two-sided, with a significance level of 0.05. Because this study was performed for exploratory purposes, statistical tests were not predefined, and multiple testing was not performed.

**Table 1:** Clinicopathological characteristics.

Characteristics	Number	%
*Age*		
Median (range)	66 (42–87)	
*Gender*		
Male	156	81.3
Female	36	18.8
*Location*		
Upper	24	12.5
Middle	76	39.6
Lower	92	47.9
*pT*		
2	30	15.6
3	156	81.3
4	6	3.1
*pN*		
0	50	26.0
1	59	30.7
2	61	31.8
3	22	11.5
*pM*		
0	175	91.1
1	17	8.9
*TNM stage*		
I	7	3.6
II	50	26.0
III	118	61.5
IV	17	8.9
*Histological grade*		
Grade 1	46	24.0
Grade 2	126	65.6
Grade 3	20	10.4

## Results

### Patient characteristics

The process of patient selection is described in Supplementary Fig. 1. Data from a total of 192 patients were included in the analysis. Table 1 details the clinicopathological characteristics. The median age of the patients was 66 (range, 42–87) years, and majority of the patients were male (81.3%). Although most of the patients had pStage II or III disease, 17 patients (8.9%) with pStage IV disease who did not have any distant organ metastases but had resectable non-regional lymph node metastases were included. None of the patients in the present study were treated with immune checkpoint inhibitors during follow-up.

### TIICs and PD-L1 expression of tumour cells

Representative macroscopic views of the cores and representative microscopic views of each stained sample and H&E stained serial sections are presented in Supplementary Fig. 2.

The number of CD8^+^ and PD-1^+^ TIICs counted by the method used in the present study was compared to that of nine randomly selected regions of interest (3 by 3) in cores from Surf, Cent, and Inv in the five initial cases. Although there was variation in the number of TIICs in the cores, particularly in those with abundant TIICs, the density of TIICs calculated based on the numbers of TIICs evaluated by these two methods were highly correlated regardless of the types of TIICs: the ρ correlation coefficients were 0.953 (*P* < 0.001) for CD8 and 0.977 (*P* < 0.001) for PD-1 (Supplementary Fig. 3).

**Fig. 1:**
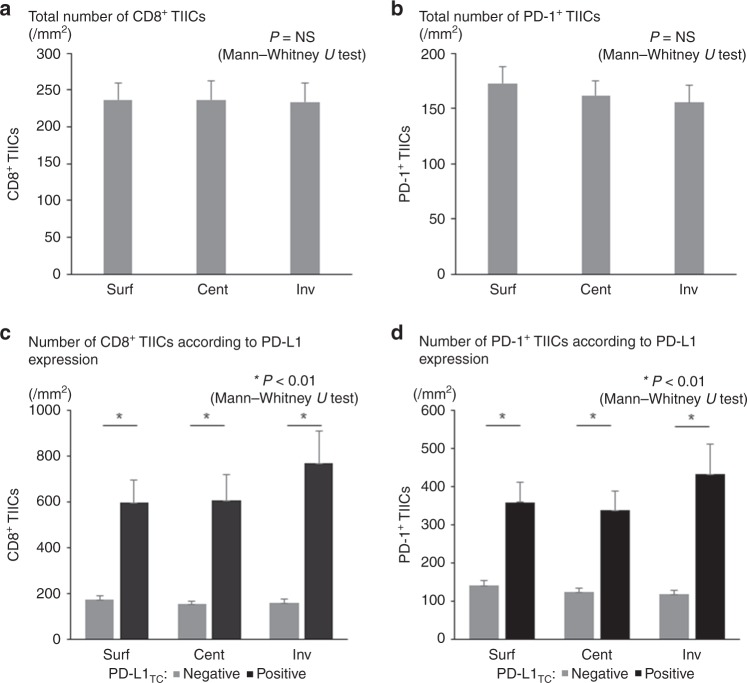
The number of TIICs according to intratumoural locations. TIIC, tumour-infiltrating immune cell. The numbers of patients with PD-L1 expression on tumour cells (PD-L1_TC_ positive) and of those with no PD-L1 expression on tumour cells (PD-L1_TC_ negative) in Surf, Cent, and Inv were 28 and 164, 35 and 157, and 23 and 169, respectively. **a** Total number of CD8^+^ TIICs. **b** Total number of PD-1^+^ TIICs. **c** Number of CD8^+^ TIICs according to PD-L1 expression. **d** Number of PD-1^+^ TIICs according to PD-L1 expression.

The mean numbers of CD8^+^ TIICs were 236.6/mm^2^ in Surf, 236.8/mm^2^ in Cent, and 234.0/mm^2^ in Inv; no significant difference in this variable was observed among each intratumoural location. The mean numbers of PD-1^+^ TIICs were 172.7/mm^2^ in Surf, 162.1/mm^2^ in Cent, and 155.7/mm^2^ in Inv; again, no significant difference was observed among each intratumoural location (Fig. 1a, b). Although patients with Grade 3 tumours showed a tendency toward higher TIIC counts for both CD8 and PD-1 compared with patients with Grade 1 and Grade 2 tumours without a statistical significance regardless of its location, no significant difference was observed among the intratumoural locations in all the subgroups according to histological grade for both CD8 and PD-1 (Supplementary Fig. 4).

**Fig. 2:**
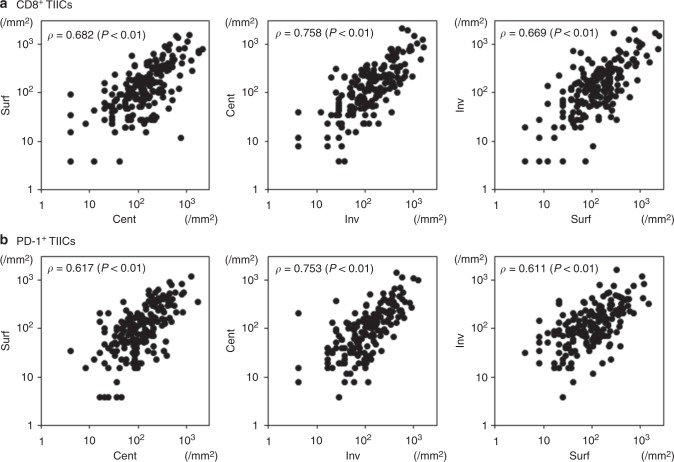
Correlation of the numbers of TIICs among intratumoural locations. TIIC, tumour-infiltrating immune cell. **a** CD8^+^ TIICs and **b** PD-1^+^ TIICs.

Figure 2 describes the correlation in the number of TIICs between each intratumoural location for CD8 and PD-1. For CD8^+^ TIICs, the correlation coefficients ρ were 0.682 (between Surf and Cent), 0.758 (between Cent and Inv), and 0.669 (between Inv and Surf). For PD-1^+^ TIICs, they were 0.617 (between Surf and Cent), 0.753 (between Cent and Inv) and 0.611 (between Inv and Surf).

The PD-L1^+^ rates were 14.6% in Surf, 18.2% in Cent, and 12.0% in Inv. Upon comparing the PD-L1^+^ rates among each intratumoural location, that in Inv was found to be significantly lower than that in Cent (*P* = 0.012) (Table 2). The numbers of CD8^+^ TIICs and PD-1^+^ TIICs were significantly higher in PD-L1^+^ tumours than in PD-L1^−^ ones in all of the intratumoural locations (Fig. 1c, d).

**Table 2:** PD-L1 positive expression rate in each intratumoral location.

	Total	Positive	%
Surf	192	28	14.6
Cent	192	35	18.2
Inv	192	23	12.0

*P* = 0.189 (Surf vs. Cent), 0.267 (Surf vs. Inv), 0.012 (Cent vs. Inv)

*Surf* surface, *Cent* center, *Inv* invasive front

### Survival analysis

**Fig. 3:**
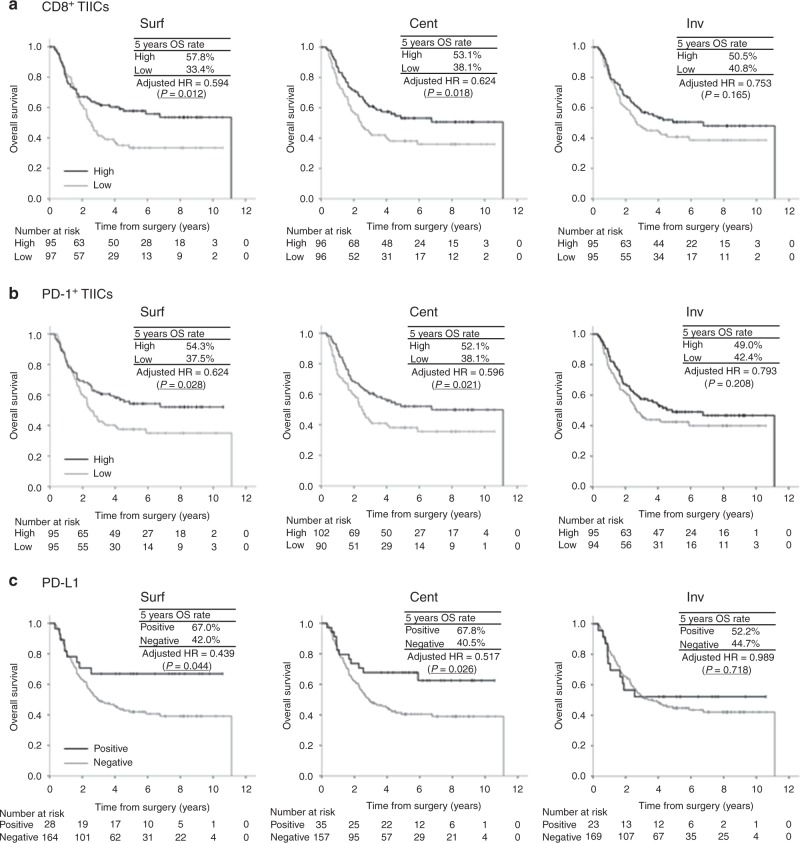
Overall survival according to the intratumoural location. TIIC, tumour-infiltrating immune cell; PD-L1_TC_, PD-L1 expression on tumour cells. **a** CD8^+^ TIICs, **b** PD-1^+^ TIICs, and **c** PD-L1_TC_.

The median follow-up time of the censored cases was 5.5 (range, 0.7–10.6) years from the date of surgery. Figure 3 describes the OS curves in terms of the numbers of CD8^+^ TIICs and PD-1^+^ TIICs and PD-L1_TC_ in each intratumoural location. Regarding CD8^+^ TIICs and PD-1^+^ TIICs, patients with high numbers of TIICs demonstrated significantly better OS than those with low numbers of TIICs in Surf and Cent, but this was not observed in comparison with those with low numbers of TIICs in Inv [CD8^+^ TIICs: adjusted HR = 0.594 (*P* = 0.012) in Surf, adjusted HR = 0.624 (*P* = 0.018) in Cent, and adjusted HR = 0.753 (*P* = 0.165) in Inv and PD-1^+^ TIICs: adjusted HR = 0.624 (*P* = 0.028) in Surf, adjusted HR = 0.596 (*P* = 0.021) in Cent, and adjusted HR = 0.793 (*P* = 0.208) in Inv]. Similarly, regarding PD-L1_TC_, patients with positivity for PD-L1_TC_ demonstrated significantly better OS than those with negativity for it in Surf and Cent, but this was not observed in comparison with those with negativity for it in Inv [adjusted HR = 0.439 (*P* = 0.044) in Surf, adjusted HR = 0.517 (*P* = 0.026) in Cent, and adjusted HR = 0.989 (*P* = 0.718) in Inv]. Patients with high and low TIICs in Inv were classified into four groups according to the number of TIICs in Inv and Surf/Cent as follows and investigated further (Supplementary Fig. 5): low in Surf and Cent and low in Inv (Surf/Cent^low^Inv^low^), low in Surf and Cent but high in Inv (Surf/Cent^low^Inv^high^), high in Surf or Cent but low in Inv (Surf/Cent^high^Inv^low^), and high in Surf or Cent and high in Inv (Surf/Cent^high^Inv^high^). As a result, for both CD8^+^ TIICs and PD-1^+^ TIICs, the five-year OS rate of Surf/Cent^low^Inv^high^ (25.4% for CD8 and 18.2% for PD-1) was much lower than that of Surf/Cent^high^Inv^high^ (54.3% for CD8 and 53.1% for PD-1). In contrast, the five-year OS rate of Surf/Cent^high^Inv^low^ (47.4% for CD8 and 49.4% for PD-1) was higher than that of Surf/Cent^low^Inv^low^ (35.7% for CD8 and 36.8% for PD-1) and rather similar to that of Surf/Cent^high^Inv^high^.

**Fig. 4:**
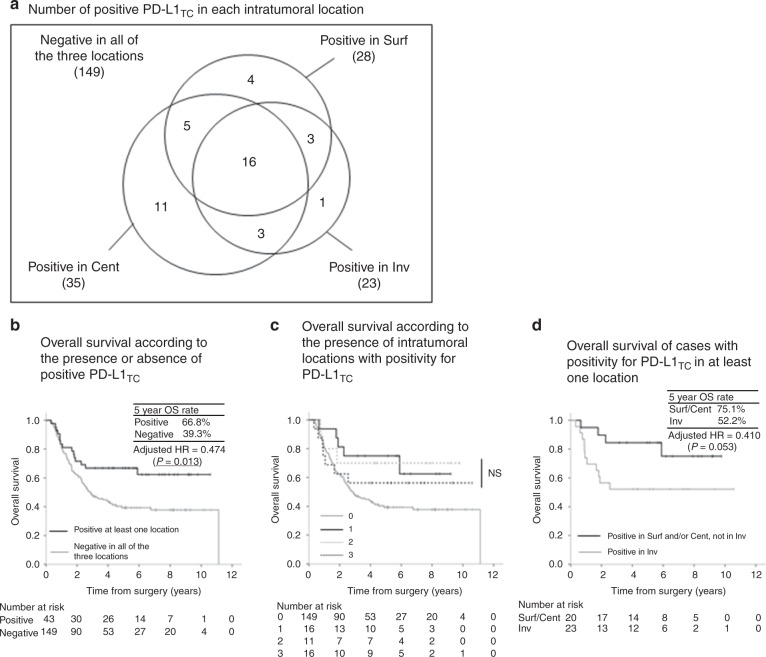
Overall survival according to PD-L1_TC_. PD-L1_TC_, PD-L1 expression on tumour cells. **a** Number of positive PD-L1_TC_ in each intratumoural location, **b** overall survival according to the presence or absence of PD-L1_TC_, **c** overall survival according to the number of intratumoural locations with positivity for PD-L1_TC_ and **d** overall survival of cases with positivity for PD-L1_TC_ in at least one location.

The relationship between OS and PD-L1_TC_ in each intratumoural location was investigated further (Fig. 4). Patients with positivity for PD-L1_TC_ in at least one intratumoural location (43 patients) demonstrated significantly better OS than those with negativity for it in all of the three intratumoural locations [adjusted HR = 0.474 (*P* = 0.013)], and the 5-year OS rate of patients with positivity for PD-L1_TC_ in at least one intratumoural location (66.8%) was comparable with that of patients with positivity for it in Surf (67.0%) and Cent (67.8%), but was higher than that of patients with positivity for it in Inv (52.2%). Among patients with positivity for PD-L1_TC_ in at least one location, there was no tendency for OS to be improved according to the increasing number of intratumoural locations with positivity for PD-L1_TC_. Among the 43 patients with positivity for PD-L1_TC_ in at least one intratumoural location, 20 patients with positivity for it in Surf and/or Cent but not in Inv demonstrated a strong tendency for better OS than 23 patients with positivity for it in Inv [adjusted HR = 0.410 (*P* = 0.053)].

We conducted survival analyses for a combination of CD8^+^ TIICs or PD-1^+^ TIICs with positivity for PD-L1_TC_ according to intratumoural locations (Supplementary Fig. 6). Only Surf demonstrated significantly better OS for both CD8^hith^PD-L1^+^ [adjusted HR = 0.394 (*P* = 0.013)] and PD-1^hith^PD-L1^+^ [adjusted HR = 0.390 (*P* = 0.012)]. Cent showed a trend for better OS for CD8^hith^PD-L1^+^ and PD-1^hith^PD-L1^+^; however, because of the small number of patients in each group, this was not statistically significant [CD8^hith^PD-L1^+^: adjusted HR = 0.657 (*P* = 0.196) and PD-1^hith^PD-L1^+^: adjusted HR = 0.608 (*P* = 0.141)]. Inv showed no statistical significance for OS and had the largest adjusted HR among the three intratumoural locations for both CD8^hith^PD-L1^+^ [adjusted HR = 0.749 (*P* = 0.392)] and PD-1^hith^PD-L1^+^ [adjusted HR = 0.675 (*P* = 0.267)].

## Discussion

Here we describe the similarities and differences in the immune status of the tumour microenvironment and its prognostic effect in different intratumoural locations in primary tumours of ESCC by assessing the numbers of TIICs and PD-L1_TC_. No standard method has been established to evaluate intratumoural heterogeneity of the immune status of tumour microenvironment although several methods, such as TMA using multiple cores from the same tumour, step sections of the same block, assessment of multiple blocks, and spiral-typed tissue cores, have been reported.^11,12,17,18^ Some studies evaluated intratumoural heterogeneity by comparing Cent and Inv irrespective of the cancer type.^17,19–21^ Based on the characteristics of gastrointestinal cancers that are present at the inside of the gastrointestinal tract, in the present study, Surf as well as Cent and Inv were evaluated.

CD8^+^ TIICs are the immune cells most frequently counted to characterise the tumour microenvironment, and abundant infiltration has repeatedly shown to be robustly related to better survival outcomes across tumour types. However, some of these cells may be inactive because of immune escape or tolerance; therefore, using activation markers or inhibitory co-stimulatory markers, including PD-1 and PD-L1, has been proposed from a functional perspective.^22^ In addition, according to several recent reports, the spatial distribution of immune cells or the spatial structure of the tumour microenvironment differs according to tumour types and molecular subtypes.^23,24^ Therefore, it is important to focus on single tumour types and conduct detailed evaluations, particularly with regard to intratumoural heterogeneity of the immune status, in addition to conducting research on multiple cancer types.

In this study, the numbers of CD8^+^ TIICs and PD-1^+^ TIICs were comparable with those reported in a previous study that examined multiple cancer types, including ESCC.^24^ As CD8^+^ lymphocytes are activated and trafficked to the tumour from the tumour-draining lymph node via antigen-presenting cells reacting to tumour-associated antigens,^25^ the comparable numbers of CD8^+^ TIICs among the three locations indicate that the immune system of the host responded similarly regardless of the location in the primary tumour. To date, discordant results regarding the distribution of CD8^+^ TIICs in the primary tumour have been reported.^18,20,26,27^ Differences in the tumour type or methodology may have contributed to this. However, in the present study, comparable infiltration of activated T cells in the tumour regardless of the location was also observed through the assessment of PD-1^+^ TIICs, a receptor of PD-L1 expressed on activated T cells,^28^ reproducing the results observed for CD8^+^ TIICs. In terms of OS according to CD8^+^ TIICs and PD-1^+^ TIICs, the similar relationship observed in Surf and Cent was not observed in Inv, although the numbers of TIICs were generally correlated among these intratumoural locations. This relationship seems to have occurred because of the existence of cases showing discordant immune cell infiltration between Surf/Cent and Inv. Furthermore, the number of TIICs in Surf and Cent was the determinant of OS regardless of the number in Inv.

The tumour microenvironment had different effects on PD-L1 expression and survival outcomes in this study. The PD-L1-positive rate was significantly lower in Inv than in Cent, whereas the numbers of TIICs were comparable and correlated between these intratumoural locations. This indicates several possibilities with regard to factors affecting PD-L1_TC_. As PD-L1_TC_ is induced by pro-inflammatory cytokines, such as IFNγ and TNFα produced by activated T cells,^29–31^ the reaction of tumour cells to the anti-tumour immune response of the host may not always be concordant among intratumoural locations. Besides, T-cell function is affected by metabolites as well as hypoxia and angiogenesis.^32^ Conversely, PD-L1_TC_ is partly regulated by an alteration of the genes of tumour cells.^33,34^ Therefore, comprehensive assessment including genomic and metabolic intratumoural heterogeneity is warranted.

Phenotypes reflecting anti-tumour response, such as abundant CD8^+^ TIICs and PD-1^+^ TIICs, and positivity for PD-L1_TC_ were related to significantly better OS in Surf and Cent, but not in Inv. Inv represents the most external portion of the tumour that is in contact with the tissue of the host, and as mentioned above, PD-L1_TC_ reflects a negative feedback reaction of tumour cells against the anti-tumour immune response. We speculate that in tumours with positivity for PD-L1_TC_ in Surf and/or Cent but not in Inv, the anti-tumour immune response acts on the tumour and the external portion has already been diminished to some extent by the attack of TIICs, as observed by a lack of PD-L1_TC,_ by the time of surgery and this results in a better prognosis. Conversely, even if the PD-L1-positivity rate itself was lower in Inv, the presence of tumours with positive PD-L1_TC_ in Inv seems to be related to resistance to anti-tumour immunity of the host and implied a poorer prognosis. In the present study, a static evaluation of the immune microenvironment of the tumour was performed at only one time point using surgically resected specimens. Therefore, there is a need to study the changes in the microenvironment over time to analyse its dynamicity. Meanwhile, considering the similarity in the number of TIICs, presence of PD-L1_TC_, and prognosis between Surf and Cent, it would be appropriate to evaluate the immune status of the tumour microenvironment using endoscopically obtained biopsy samples from Surf. Also, during examination of surgically resected samples, the focus should be on Surf and Cent and not on Inv.

The present study has some limitations. First, although PD-L1_TC_ was used to assess the response of the tumour cells to anti-tumour immunity, the optimal PD-L1 assessment method has not been established yet. Several methods, such as assessing the expression of tumour cells, stromal cells, and both tumour and stromal cells or using different thresholds for positivity, have been tested in clinical trials.^35–37^ Second, we focused on the expression of PD-L1 as an inhibitory reaction of tumour cells to anti-tumour immune function and cytotoxic T cells, which attack tumour cells directly. However, there are multiple types of immune cells, such as regulatory T cells, dendritic cells, and myeloid cells, in the tumour microenvironment, and the relationship between these cells and clinical outcomes was not addressed in this study. Third, TIICs in the four areas containing the highest number of TIICs were quantified manually in this study. Although this method has been used as a standard method^15,16,38^ and we confirmed that this method could represent the number of TIICs in each core, with recent advances in digital quantification, a new technology can be applied in future studies for the digital quantification of entire cores.^24,39^ Lastly, although the different survival impact of the tumour microenvironment in different intratumoural locations was consistent with regard to multiple factors, such as PD-L1_TC_ and numbers of CD8^+^ and PD-1^+^ TIICs, evaluation—preferably a prospective study—in a different cohort that includes other biomarkers for comprehensive analysis is warranted to confirm the results of this study.

## Conclusion

Despite the comparable numbers of TIICs and their correlated degrees of infiltration in different intratumoural locations, PD-L1 expression and the relationship between the immune microenvironment and survival outcomes differed according to the intratumoural location of ESCC. This study shows that the immune microenvironments of Surf and Cent have clinical impact.

## Supplementary information

Supplementary files

## Data Availability

The datasets used during the present study are available from the corresponding author on reasonable request.
