# Supplementary material for: Relationship between the immune microenvironment of different locations in a primary tumour and clinical outcomes of oesophageal squamous cell carcinoma
Source: Br J Cancer. 2019 Nov 25;122(3):413–20. doi: 10.1038/s41416-019-0622-3 (PMC7000821; doi:10.1038/s41416-019-0622-3)
Supplement: Supplementary file 1 — Supplementary files [file 41416_2019_622_MOESM1_ESM.docx]

**Supplementary Figure 1. The process of patient selection.**


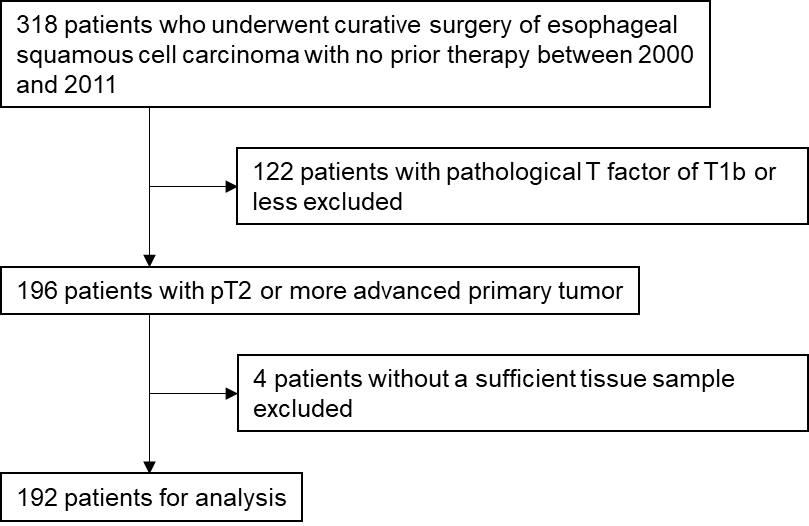


**Supplementary Figure 2. Representative views of tissue samples.**

**A. Representative macroscopic view of collecting cores. A 2.0-mm-diameter tumor core was obtained from the surface (Surf), the center (Cent), and the invasive front (Inv) of the primary tumor. Dotted line, tumor area.**


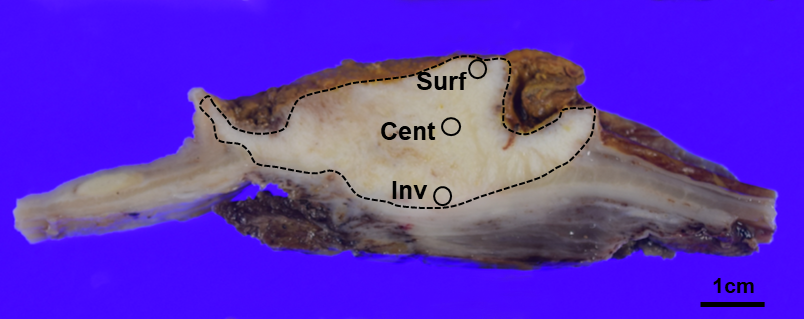


**B. Representative microscopic view of each staining on serial sections**


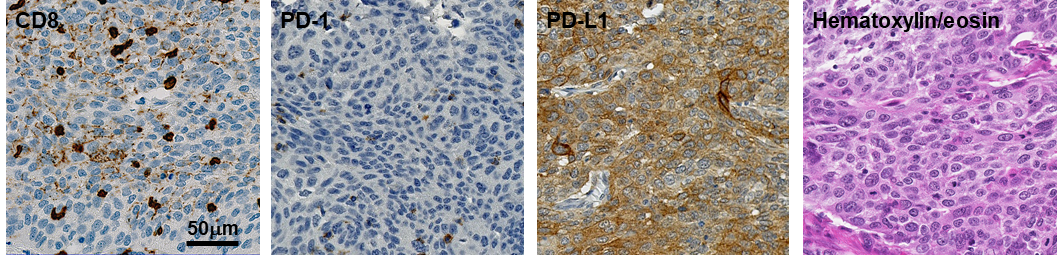


**Supplementary Figure 3. Validation of the method used in the present study.**

**A. Distribution of the numbers of TIICs in nine randomly selected regions in cores from Surf, Cent, and Inv. X-axis: Surf, Cent, and Inv from the left in each case. The boxes describe the first and third quartiles, with the median (bar inside the box), average (x), and whiskers describing the minimum and maximum values of the nine regions in each core.**


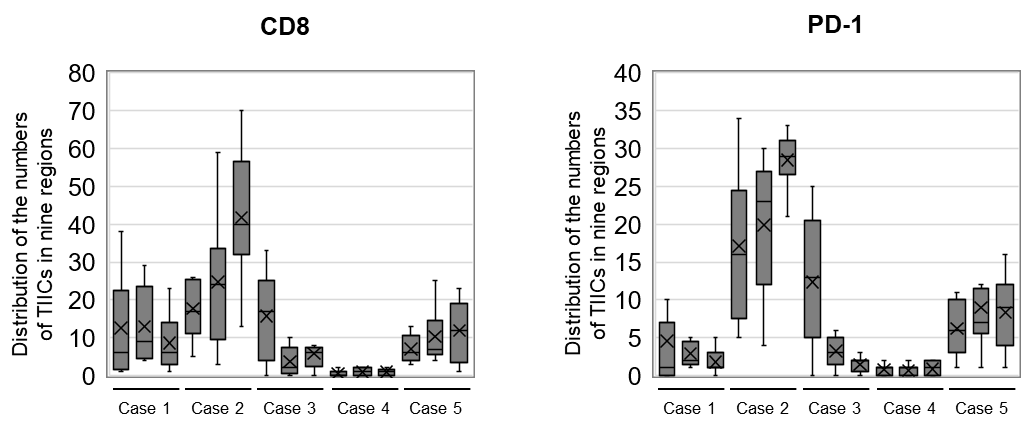


**B. Correlation of the numbers of TIICs between the four abundant areas and nine randomly selected areas (Surf: triangle, Cent: circle, Inv: square).**


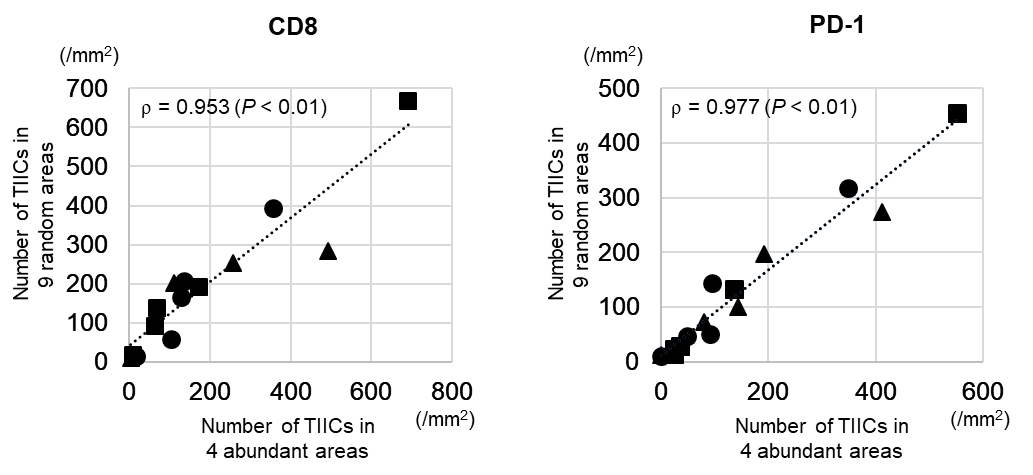


**Supplementary Figure 4. Comparison of the number of TIICs among each intratumoral location according to histological grade: Grade 1 (n = 46), Grade 2 (n = 126), Grade 3 (n = 20).**


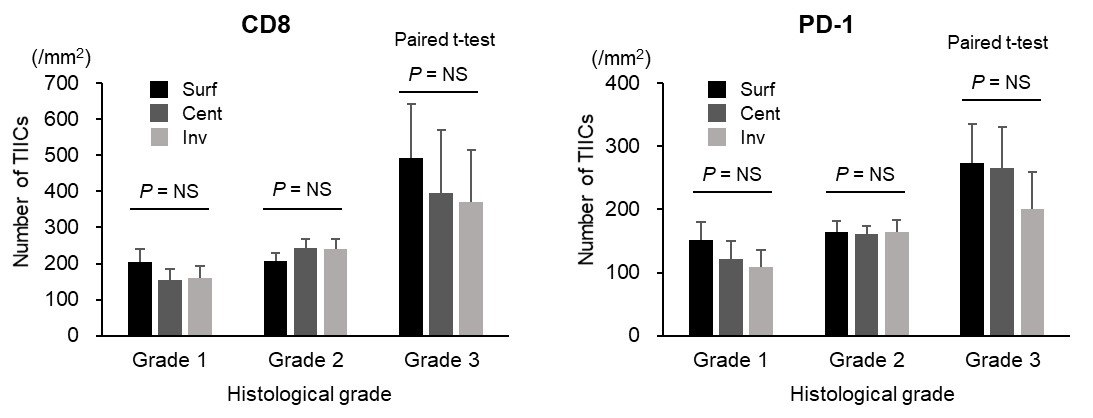


**Supplementary Figure 5. Overall survival according to tumor-infiltrating immune cells in relation to Surf/Cent and Inv.**


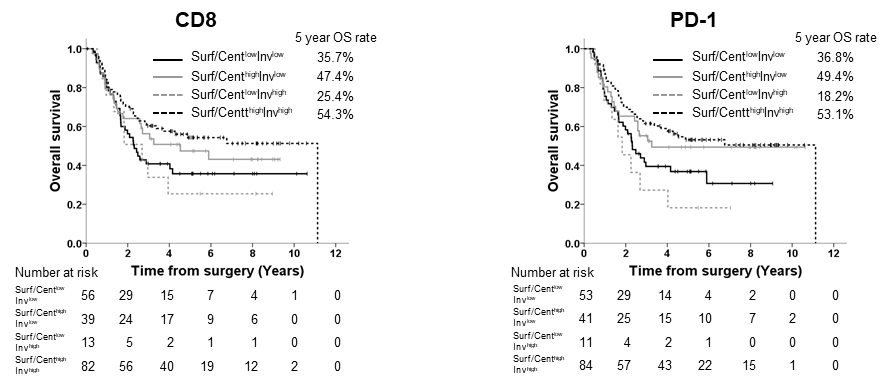


**Supplementary Figure 6. Overall survival according to intratumoral location. TIIC, tumor-infiltrating immune cell; PD-L1_TC_, PD-L1 expression on tumor cells.**

**A. CD8^high^/PD-L1**


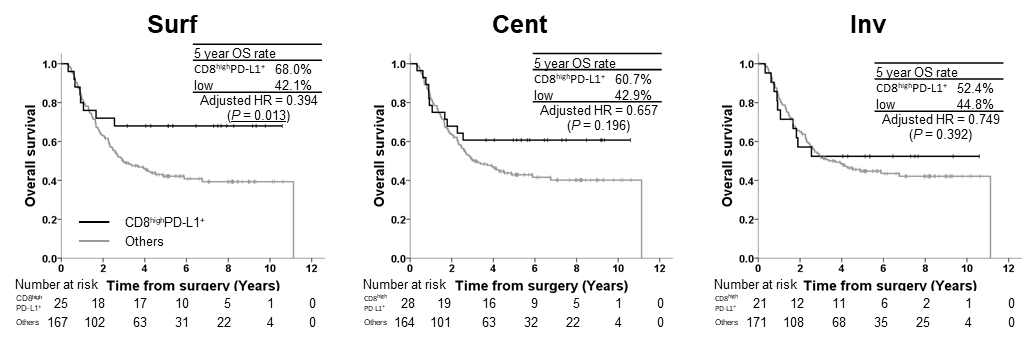


**B. PD-1^high^/PD-L1**


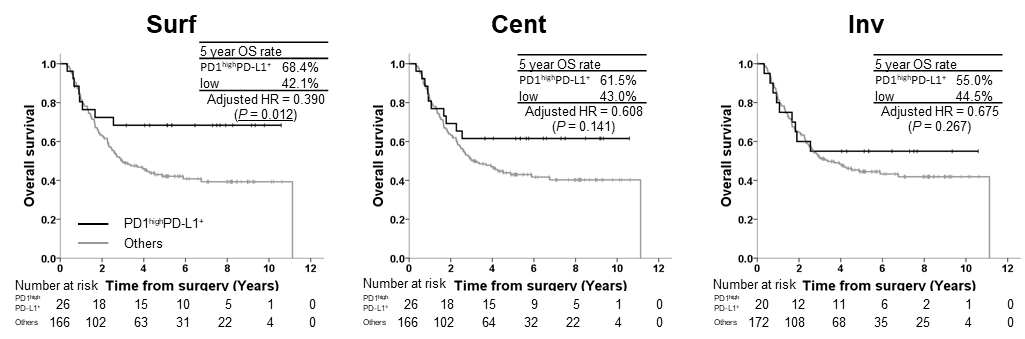


| Supplementary Table 1. Antibodies and immunohistochemical assays | | | | | | |  |  |
| --- | --- | --- | --- | --- | --- | --- | --- | --- |
| Marker | Source | Type | Clone | Procedure | Dilution | Antigen retrieval | Visualization | Manufacturer |
| CD8 | Rabbit | Monoclonal | SP57 | Autostainer | Ready-to-use | Heat (95°C, 64 min.), | ultra View Universal DAB Detection Kit | Ventana (Tucson, USA) |
|  |  |  |  |  |  | CC1: EDTA buffer (pH 8.5) |  |  |
| PD-1 | Mouse | Monoclonal | EH33 | Manual | 1:200 | M/W (95°C, 20 min.), | Standard DAB procedure | Cell Signaling Technology (Cambridge, UK) |
|  |  |  |  |  |  | EDTA buffer (pH 9.0) |  |  |
| PD-L1 | Rabbit | Monoclonal | E1L3N | Manual | 1:400 | M/W (95°C, 10 min.), | Standard DAB procedure | Cell Signaling Technology |
|  |  |  |  |  |  | Citrate buffer (pH 6.0) |  |  |
| Abbreviations: M/W, microwave; EDTA, ethylenediaminetetraacetic acid; DAB, diaminobenzidine | | | | | | | | |
